# Toxin release mediated by the novel autolysin Cwp19 in *Clostridium difficile*

**DOI:** 10.15698/mic2018.09.648

**Published:** 2018-08-10

**Authors:** Imane El Meouche, Johann Peltier

**Affiliations:** 1 Dunlop lab, Boston University, Boston, Massachussets, USA.; 2 Laboratoire Pathogenese des Bacteries Anaerobies, Institut Pasteur, Paris, France.; 3 Universite Paris-Diderot, Universite Sorbonne Paris Cite, Paris, France.

**Keywords:** Clostridium difficile, peptidoglycan hydrolase, autolysin, lytic transglycosylase, Cwp19, toxins secretion, glucose

## Abstract

*Clostridium difficile*, also known as *Clostriodioides difficile*, is a Gram positive, spore-forming bacterium and a leading cause of antibiotic-associated diarrhea in nosocomial environments. The key virulence factors of this pathogen are two toxins, toxin A and toxin B, released from the cells to the gut and causing colonic injury and inflammation. Although their mechanism of action is well known, the toxins A and B have no peptide signals and their secretion mechanisms involving the holin-like protein TcdE and autolysis are still under active investigation. Autolysis is primarily mediated by peptidoglycan hydrolases, an important group of enzymes that cleave covalent bonds in the cell wall peptidoglycan. Peptidoglycan hydrolases are essential for peptidoglycan remodeling but most of them also have the potential to lyse the cells under various conditions. In a recent report by Wydau-Dematteis *et al.* (MBio 9(3): e00648-18), we characterized a novel peptidoglycan hydrolase Cwp19 in *C. difficile*. Importantly, Cwp19 mediates toxins secretion in a glucose-dependent fashion suggesting a potential role in *C. difficile* pathogenesis. Peptidoglycan hydrolases are not very well characterized in *C. difficile* despite the important role of these enzymes in cell division and sporulation as shown in model organisms like *Bacillus subtilis*. In addition, these enzymes can be implicated in pathogenicity as exemplified by the release of pneumococcal virulence factors.

## INTRODUCTION

*Clostridium difficile* is a leading cause of infectious diarrhea in hospitalized patients in Europe, North America and Australia. *C. difficile* infections are extremely costly to treat and represent a substantial economic burden. *C. difficile* infection is associated with a wide range of clinical manifestations ranging from mild diarrhea to more severe pseudomembranous colitis and death. The most recognized risk of *C. difficile* infection is antibiotic therapy, perturbing the internal microflora and making it vulnerable to *C. difficile* spores germination, growth and colonization. In addition, the relapse of *C. difficile* infection is a major issue making the treatment less effective.

*C. difficile* pathogenesis depends on the production of two large clostridial toxins, TcdA and TcdB, which are responsible for the symptoms associated with *C. difficile* infection. Both toxins are Rho GTPase-glucosylating cytotoxins that disrupt the actin cytoskeleton of intestinal epithelial cells, cause tissue damage, necrosis and inflammation. TcdA and TcdB are released from the cells to the gut without an identifiable secretion signal. The mechanism for their release was not fully understood until recently when Wydaw-Dematteis *et al*. characterized the cell surface protein Cwp19, a peptidoglycan lytic enzyme involved in autolysis of vegetative cells of *C. difficile* and toxin release in response to specific environment conditions.

In this paper, a proteomic analysis combining the use of zymography, SDS-PAGE, and liquid chromatography-tandem mass spectrometry identified Cwp19 as a major peptidoglycan-degrading enzyme. Cwp19 harbored a C-terminal cell wall binding motif and an N-terminal domain belonging to glycosyl hydrolase-like family, a domain widely distributed in Gram-positive and negative bacteria as Firmicutes, Actinobacteria, Bacteroidetes, Cyanobacteria, and Proteobacteria. To our knowledge, a peptidoglycan hydrolase has not been attributed to this domain before. Data in this paper showed Cwp19 as a peptidoglycan lytic enzyme having lytic transglycosylase specificity and the tandem mass spectrometry fragmentation of peptidoglycan digestion products generated by Cwp19 identified the typical anhydromuropeptides resulting from this activity. Few lytic transglycosylases have been characterized in Gram-positive bacteria, and they were mostly involved in sporulation or germination. However, Cwp19 did not significantly impact the sporulation of *C. difficile* and does not show homology to other previously identified lytic transglycosylases.

The data in the paper showed no obvious role for Cwp19 in cell division and inactivation of the corresponding gene did not impair cell growth. However, Cwp19 seemed to be a key player in cell autolysis triggered at the beginning of the stationary phase. This result is supported by transmission electron microscopy showing that the cell wall of cells lacking Cwp19 was intact and well defined, and almost no lysed cells were observed compared to the wild type. Furthermore, toxins A and B synthesis in *C. difficile* happens largely in stationary phase. Interestingly, although the absence of Cwp19 did not affect toxin production, toxin release into the extracellular medium was significantly delayed confirming the critical role of bacteriolysis in this process. Cwp19 is conserved among *C. difficile* sequenced isolates and it is one of the cell wall proteins present at the cell surface *in vivo*, which makes it an important candidate in *C. difficile* pathogenesis. This study was conducted in an historic strain but it will be highly relevant to expand the analysis of Cwp19 function to epidemic strains of *C. difficile.*

**Figure 1:**
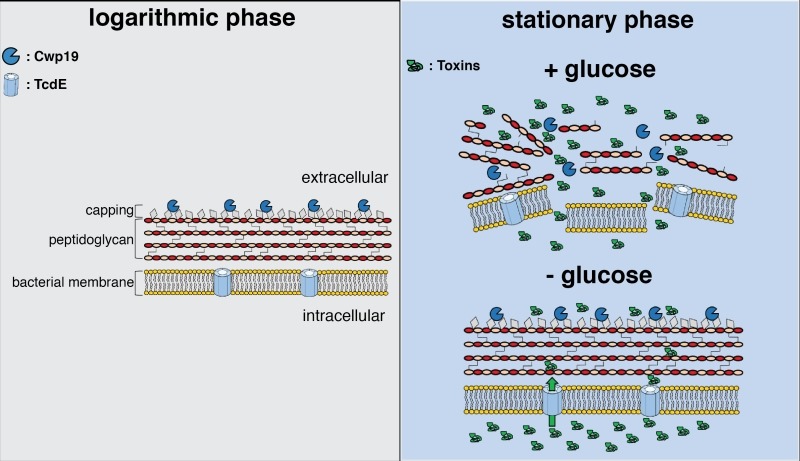
FIGURE 1: Illustration showing a model for Cwp19 activity and toxin release. Cwp19 is associated to the cell wall during logarithmic phase and active but is unable to induce cell lysis, due to peptidoglycan inaccessibility. We propose that the inability of Cwp19 to exert lytic activity is due to a protective capping of the peptidoglycan. Upon entry into stationary phase, *C. difficile* cells produce toxins and two different mechanisms mediate their release into the extracellular environment. These mechanisms seem to depend on the presence or absence of glucose in the environment. In presence of glucose, modifications in the cell wall composition will result in the exposure of uncapped peptidoglycan, making it available for Cwp19 to initiate cell wall degradation. This will lead to cell lysis and release of the intracellular content, including toxins, from the cytoplasm. In absence of glucose, cells remain protected from the lytic activity of Cwp19. High levels of toxins are produced in this condition and they are secreted through another mechanism involving the holin-like protein TcdE.

The impact of Cwp19 on stationary phase autolysis was shown in media containing glucose. The role of the holin-like protein TcdE in toxin release has been largely discussed in the literature. All the experiments previously realized to confirm TcdE as a mediator of toxins secretion were realized in a medium lacking glucose. Cwp19 role in cell autolysis and toxin release in absence of glucose was investigated in this study and interestingly a different phenomenon was observed. Significant toxin release was still observed during the early stationary phase although Cwp19 was not able to trigger lysis. It is important to note that the *cwp19* gene was expressed and the enzyme was active. This result is intriguing and it shows how *C. difficile* can combine two mechanisms of toxins release that depend on glucose, i.e. bacteriolysis and TcdE-mediated secretion (Figure 1). This also opens perspectives on a wider set of questions. More is needed to know when Cwp19 would be a preferred mechanism for *C. difficile* to release toxins, is it only in presence of glucose when the toxins levels are not too high? Of note, toxin synthesis is repressed in presence of glucose. Further studies will be performed to evaluate the response of Cwp19 to nutritional stresses. Does Cwp19 absence influence *C. difficile* pathogenicity *in-vivo*? Testing the impact of *cwp19* inactivation in a hamster infection model and a mouse colonization model will answer this question. Additional elements will also be needed to understand the interplay between TcdE mediated release and bacteriolysis in an environment mimicking as much as possible the conditions in the gut. Nutrients availability during *C. difficile* infection changes with the trajectory of infection. Future studies will aim to decipher the asynchrony between TcdE and Cwp19 mediated toxin synthesis. Single-cell studies should be performed in nutritional stress conditions to study if a sub-population of cells diversifies its mechanisms of toxin release in conditions mirroring different stages of *C. difficile* pathogenesis and dissemination.

In addition to the glucose-dependent role in toxin release and autolysis, the mechanism and timing of when Cwp19 triggers autolysis are intriguing. First, Cwp19 activity was not detected on peptidoglycan extracted from *C. difficile*, the hydrolysis specificity was established when Cwp19 was incubated with purified peptidoglycan from *Micrococcus lysodeikticus*. Peptidoglycan is a complex polymer with structural modifications that can regulate peptidoglycan hydrolases activity. In *C. difficile*, one such modification is the extensive deacetylation of *N*-acetylglucosamine of the glycan chains. It is likely that this feature might prevent the recognition and digestion of *C. difficile* peptidoglycan by Cwp19, leading to only few cleavage sites. The validation of this hypothesis will require assessing Cwp19 activity on modified “re-acetylated” peptidoglycan of *C. difficile*. The genome of *C. difficile* encodes at least four putative deacetylases targeting the vegetative peptidoglycan. Inactivation of one or several of the corresponding genes should result in a significant proportion of *N*-acetylglucosamine in the glycan chains.

In addition, Cwp19 is mainly expressed during active growth, however, it is implicated in stationary phase lysis only. This result hints that cell wall modifications seem to be essential in order to permit Cwp19 autolysis. All strains of *C. difficile* produce the teichoic-like acids PSII and the lipoteichoic-like acids PSIII. The display of these secondary cell wall polymers at the cell surface might protect the cells from lysis by capping the peptidoglycan (Figure 1). Changes in the abundance and/or the composition of these polymers would then occur during the stationary phase in presence of glucose that would facilitate the access of Cwp19 to its substrate and trigger the initiation of cell wall digestion. Further studies would be essential to understand if and how the secondary cell wall polymers regulate Cwp19 activity but also how Cwp19 is essential in stationary phase autolysis despite having limited cleavage activity on *C. difficile* peptidoglycan.

At some point in late stationary phase, the cells will lyse independently of Cwp19, reflecting the implication of other autolysins. Besides Cwp19, sequence comparison by database analysis identified thirty putative peptidoglycan-degrading enzymes in *C. difficile*. Among them, seven are under transcriptional control of a sporulation-specific Sigma-factor and/or are homologous to enzymes specifically involved in the sporulation or germination process in *B. subtilis*. This still leaves twenty-three peptidoglycan lytic enzymes possibly active on the vegetative cell wall peptidoglycan, raising the question of their respective physiological functions in the different aspects of peptidoglycan metabolism. None of the latter enzymes have been characterized to date and it is difficult to understand why *C. difficile* needs such a large set of peptidoglycan hydrolases. Moreover, the multiplicity of these enzymes strongly suggests functional redundancy and makes the study of their physiological function a challenging task. Further work will be done on identifying other major peptidoglycan hydrolases in *C. difficile*, knowing their potential importance in the physiology and virulence of this pathogen.

Finally, Cwp19 seems to be an attractive drug target to prevent toxin release during *C. difficile* infection. Deciphering both mechanisms of its activity and the nature of the signals controlling it are promising to find new therapeutic approaches targeting *C. difficile* cell autolysis before toxin production.

